# Dinosaur Speed Demon: The Caudal Musculature of *Carnotaurus sastrei* and Implications for the Evolution of South American Abelisaurids

**DOI:** 10.1371/journal.pone.0025763

**Published:** 2011-10-17

**Authors:** W. Scott Persons, Philip J. Currie

**Affiliations:** Department of Biological Sciences, University of Alberta, Edmonton, Alberta, Canada; Raymond M. Alf Museum of Paleontology, United States of America

## Abstract

In the South American abelisaurids *Carnotaurus sastrei, Aucasaurus garridoi*, and, to a lesser extent *Skorpiovenator bustingorryi*, the anterior caudal ribs project at a high dorsolateral inclination and have interlocking lateral tips. This unique morphology facilitated the expansion of the caudal hypaxial musculature at the expense of the epaxial musculature. Distinct ridges on the ventrolateral surfaces of the caudal ribs of *Aucasaurus garridoi* are interpreted as attachment scars from the intra caudofemoralis/ilio-ischiocaudalis septa, and confirm that the *M. caudofemoralis* of advanced South American abelisaurids originated from a portion of the caudal ribs. Digital muscle models indicate that, relative to its overall body size, *Carnotaurus sastrei* had a substantially larger *M. caudofemoralis* than any other theropod yet studied. In most non-avian theropods, as in many extant sauropsids, the *M. caudofemoralis* served as the primary femoral retractor muscle during the locomotive power stroke. This large investment in the *M. caudofemoralis* suggests that *Carnotaurus sastrei* had the potential for great cursorial abilities, particularly short-burst sprinting. However, the tightly interlocking morphology of the anterior caudal vertebrae implies a reduced ability to make tight turns. Examination of these vertebral traits in evolutionary context reveals a progressive sequence of increasing caudofemoral mass and tail rigidity among the Abelisauridae of South America.

## Introduction

When first described by Bonaparte *et al*. in 1990 [1], the holotype of *Carnotaurus sastrei* (MACN-CH 894) revealed many puzzling adaptations in both the appendicular and axial skeleton that were previously unseen in theropods. *C. sastrei* established Abelisauridae as a unique clade of carnivorous dinosaurs, evidently separated from all other known theropod groups by a large evolutionary rift [1]. Currently, Abelisauridae is best known for the small horns and other cranial ornamentations common to most of its members. *C. sastrei* is the most advanced member of Abelisauridae, with a pair of robust conical horns that extend devilishly from the frontals. However, the most unusual skeletal adaptations of *C. sastrei* and its close relatives occur not in the skull, but in the tail.

**Figure 1:**
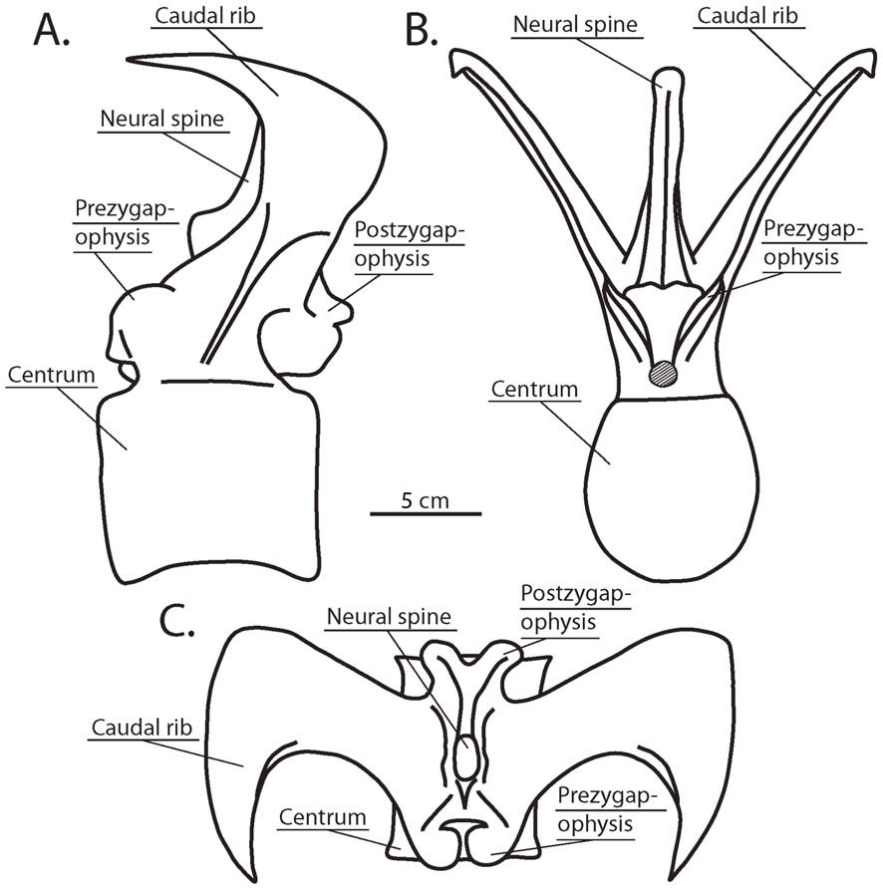
Typical anterior caudal vertebral morphology of *Carnotaurus sastrei* (MACN-CH 894). Restored illustration of caudal vertebra 6 in (A) left lateral view, (B) anterior view, and (C) dorsal view.

**Figure 2:**
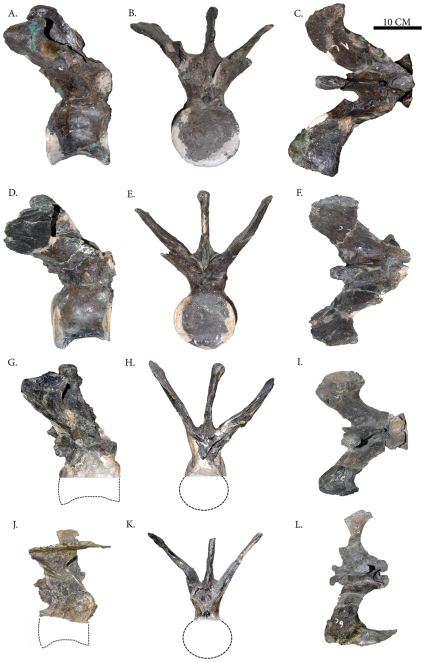
Select caudal vertebra of *Carnotaurus sastrei* (MACN-CH 894). (A, B, C) Caudal vertebra 1 in right lateral, anterior, and dorsal view, respectively – note: caudal rib tips not fully preserved. (D, E, F) Caudal vertebra 2 in right lateral, anterior, and dorsal view, respectively; note: caudal rib tips not fully preserved. (G, H, I) Caudal vertebra 5 in right lateral, anterior, and dorsal view, respectively – note: centrum and caudal rib tips not fully preserved. (J, K, L) Caudal vertebra 6 in right lateral, anterior, and dorsal view, respectively; note: centrum and left caudal rib tip not fully preserved.

The preserved tail vertebrae of MACN-CH 894 have caudal ribs that are posteriorly inclined, dorsally angled, and often exceed the neural spines in absolute height [1]. The tips of the caudal ribs are flattened and expanded with anteriorly-projecting half-crescent-shaped anterior edges and rounded posterior edges (Figs. 1, 2). Since the initial description of *C. sastrei*, many aspects of this bizarre caudal morphology have been reported in other South American abelisaurids, including *Aucasaurus garridoi* [2], *Ilokelesia aguadagradensis* [3], and *Skorpiovenator bustingorryi* [4]. Abelisaurids in Madagascar and Southern Asia have consistently shown an absence of this unusual morphology. In the Malagasy genus *Majungasaurus*, for which a largely complete caudal series is known, the general proportions of the caudal osteology do not differ dramatically from those of most other large-bodied non-coelurosaurian theropods. The anterior caudal ribs of *Majungasaurus crenatissimus* project predominantly transversely, with only a slight ventral inclination, and lack specialized caudal rib tips [5].

The prominent lateral projections of the caudal vertebrae are here referred to as “caudal ribs,” in preference to the term “transverse processes”. Conclusive osteological evidence supporting one terminology over the other is currently lacking among theropods. Although the latter term has become conventional within much of the paleontological literature, the accuracy of the former term has been established in developmental studies on modern sauropsids, including crocodilians [6]–[9]. As here used, the term “caudal rib” should also not be confused with the arguments made by Carrano *et al*. [10] (later discussed), which suggests that both caudal ribs and caudal transverse processes were present in some abelisaurids.

Beginning with the original description of *C. sastrei*, the potential athleticism of abelisaurids has been the subject of speculation and debate. Based on the proportions of the hind limbs, Bonaparte *et al*. [1] suggested that *C. sastrei* was among the most cursorial of the large-bodied theropods, and Mazzetta *et al*. [11] supported this inference. However, the subsequent discovery of complete hind limbs in the type specimen of the closely related *Aucasaurus garridoi* showed that the length ratio of the tibia/femur was likely not a high as Bonaparte *et al*. [1] anticipated [2]. In *Majungasaurus crenatissimus*, the total tibia-femur length is notably short compared to other similarly sized theropods [12], suggesting that *Majungasaurus crenatissimus* was comparably slow. The hind limbs of the Indian abelisaurid *Rajasaurus narmadensis* and the South American abelisaurid *Ekrixinatosaurus novasi* were proportioned similarly to *Majungasaurus crenatissimus*, while the legs of *C. sastrei* and *Aucasaurus garridoi* were relatively longer and more gracile [12]–[14].

Consideration of the novel caudal osteology of *C. sastrei* and its South American kin is potentially relevant to the discussion on abelisaurid cursoriality, because the tails of most non-avian theropods, like the tails of most other non-avian sauropsids, were the origin sites for the primary hind-limb retractor muscle: the *M. caudofemoralis* [15]–[17]. The *M. caudofemoralis* inserts onto the fourth trochanter of the femur, and contraction of the *M. caudofemoralis* swings the femur posteriorly. Electromyographic studies on extant crocodiles have shown that the *M. caudofemoralis* is active during locomotion [18], and, because of its size, the muscle is assumed to contribute the majority of force to the hind limb's locomotive power stroke [17], [19]. Recent advances have been made in the study of dinosaur caudal musculature and in the struggle to estimate the size of individual caudal muscles from osteological correlates [20]–[23]. It is now recognized that the dimensional extents of the various caudal muscle sets were not limited to those of the caudal skeleton [21]–[23]. Persons and Currie [22] outlined a simple method for conservatively estimating the mass of the *M. caudofemoralis* and other major caudal muscles based on caudal osteology, and concluded that the size of the *M. caudofemoralis* of most non-avian theropods was proportionately larger and more laterally extensive than in modern crocodiles and lizards. Here, the same techniques are applied to a digital reconstruction of the caudal skeleton of *C. sastrei* (with posterior portions modeled after those of more complete closely related theropods).

### Institutional Abbreviations

**BHI,** Black Hills Institute of Geological Research, Hill City, South Dakota, USA; **FMNH**, Field Museum of Natural History, Chicago, Illinois, USA; **LACM**, Natural History Museum of Los Angeles County, Los Angeles, California, USA; **MACN-CH**, Museo Argentino de Ciencias Naturales “B. Rivadavia,” Coleccion Chubut, Argentina; **MCF-PVPH**, Museo Municipal “Carmen Funes”, Paleontologia de Vertebrados, Plaza Huincul, Argentina; **USNM**, Smithsonian Institution, National Museum of Natural History, Washington, District of Colombia, USA; **TMP**, Royal Tyrrell Museum of Palaeontology, Drumheller, Alberta, Canada.

## Results and Discussion

### Reconstruction Results

**Figure 3:**
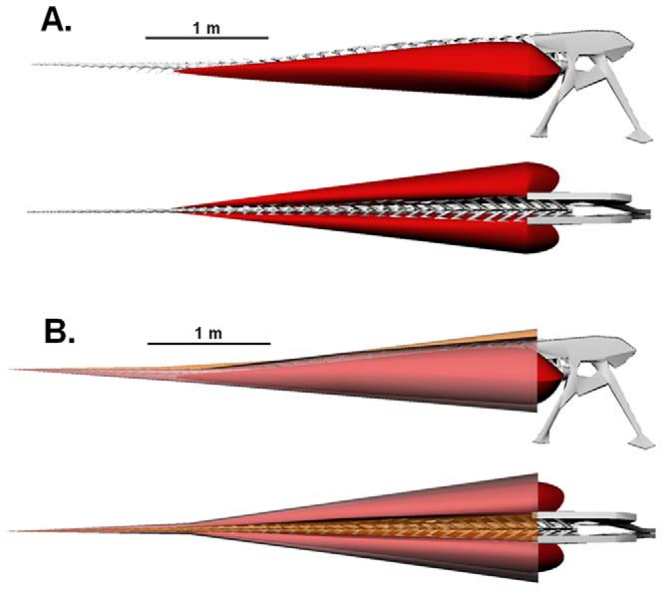
Lateral and dorsal views of the robustly modeled tail of *Carnotaurus sastrei* (MACN-CH 894). (A) Digital reconstruction of the caudal and pelvic skeleton with *M. caudofemoralis longus* (red). (B) Complete digital reconstruction, with epaxial musculature (orange) and *M. ilio-ischiocaudalis* (pink) added.

**Figure 4:**
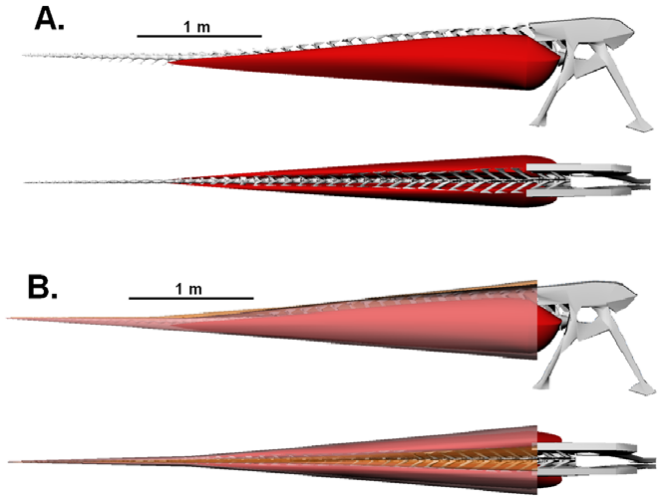
Lateral and dorsal views of the conservatively modeled tail of *Carnotaurus sastrei* (MACN-CH 894). (A) Digital reconstruction of the caudal and pelvic skeleton with *M. caudofemoralis longus* (red). (B) Complete digital reconstruction, with epaxial musculature (orange) and *M. ilio-ischiocaudalis* (pink) added.

**Figure 5:**
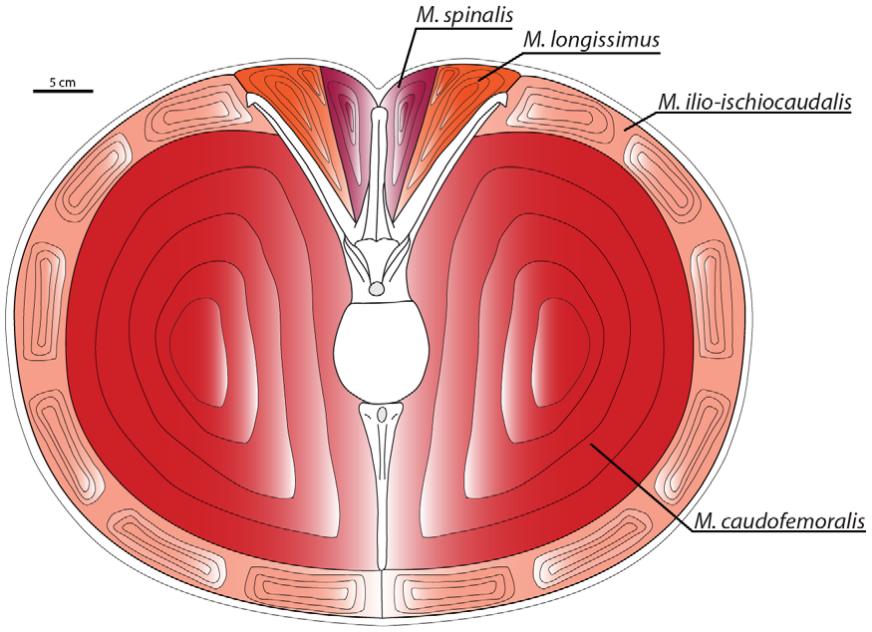
Cross-section through the tail of *Carnotaurus sastrei* showing caudal vertebra 6 and accompanying musculature. Note: the cross-section is an anatomical abstraction and depicts the neural arch and chevron in the same vertical plane.

It is apparent from simple observation of the fossil specimens of both *Carnotaurus sastrei* and *Aucasaurus garrido* that the dorsal tilt of the caudal ribs and the insertion of the *M. caudofemoralis* onto the lateral surfaces of the caudal ribs permitted the dorsal expansion of the *M. caudofemoralis,* even past the point of mediolateral overlap with the *M. longissimus*. The results of the digital modeling are summarized in Table 1 and indicate a substantial investment in hypaxial vs. epaxial musculature (Figs. 3,4,5). The calculated mass of the *M. caudofemoralis* is particularly large, estimated to range from 111–137 kg for each hind limb. In Persons and Currie [22], the methods used here to create the conservative muscle model were tested on a range of modern long-tailed sauropsids and were found to consistently underestimate true *M. caudofemoralis* mass, but to within 1–6% of the true value. The overall muscle to bone proportions of the robust model exceed the typical range reported by Allen *et al*. [21] for a variety of modern lizards. The true mass of the *M. caudofemoralis* of *C. sastrei*, therefore, likely lies within this range, but probably not at either extreme. Compared with the other muscles, the estimated mass of the *M. ilio-ischiocaudalis* varied the most between the conservative and robust models. This is because, in the robust reconstruction, both the absolute thickness of *M. ilio-ischiocaudalis* was increased and, because the *M. caudofemoralis* was expanded laterally, the elliptical path of the *M. ilio-ischiocaudalis* was also increased.

**Table 1:** Mass estimation results from the conservative and robust models of *Carnotaurus sastrei* (results are presented for left and right muscle sets combined).

	M. spinalis	M. longissimus	M. ilio-ischiocaudalis	M. caudofemoralis
**Conservative Model**	7000 g	15000 g	63000 g	222000 g
**Total tail muscle mass: 307000 g**	2.30%	4.90%	20.50%	72.30%
**Total body mass: 1500000 g** [**39**]	0.50%	1.00%	4.20%	14.80%
**Robust Model**	11000 g	24000 g	106000 g	273000 g
**Total tail muscle mass: 414000**	2.70%	5.80%	25.60%	65.90%
**Total body mass: 1500000 g** [**39**]	0.70%	1.60%	7.10%	18.20%

Comparisons of the conservative muscle mass estimations with those obtained using the same methods for other theropods are given in Table 2, and confirm that *C. sastrei* had an exceptionally large investment in the *M. caudofemoralis* – estimated to be greater relative to overall body size than that previously calculated for any other theropod.

**Table 2:** Estimated conservative caudal muscle masses of *Carnotaurus sastrei* and other theropods (results are presented for left and right muscle sets combined).

	M. spinalis	M. longissimus	M. ilio-ischiocaudalis	M.caudofemoralis
***Carnotaurus sastrei*** ** MACN-CH 894**	7000 g	15000 g	63000 g	222000 g
**Total tail muscle mass: 307000 g**	2.30%	4.90%	20.50%	72.30%
**Total body mass: 1500000 g** [**11**]	0.50%	1.00%	4.20%	14.80%
***Ornithomimus edmontonicus*** ** TMP 95.11.001**	860 g	2440 g	5050 g	9890 g
**Total tail muscle mass: 18240g**	4.70%	13.40%	27.70%	54.20%
**Total body mass: 150000 g** [**22**]	0.60%	1.60%	3.40%	6.60%
***Gorgosaurus libratus*** ** TMP 91.36.500**	3900 g	6900 g	10300 g	17300 g
**Total tail muscle mass: 38300 g**	10.20%	18.00%	26.90%	45.20%
**Total body mass: 400000 g** [**22**]	1.00%	1.70%	2.60%	4.30%
***Tyrannosaurus rex*** ** BHI 3033**	65200 g	154200 g	159400 g	522200 g
**Total tail muscle mass: 901000 g**	7.20%	17.10%	17.70%	58.00%
**Total body mass: 5622000 g** [**39**]	1.20%	2.70%	2.80%	9.30%

*Gorgosaurus libratus*, *Ornithomimus edmontonicus*, and *Tyrannosaurus* rex estimations are taken from Persons and Currie 2011 [6].

### Functional Implications

The models created in this study are largely based on both *Carnotaurus sastrei* and *Aucasaurus garrido*. For the sake of simplicity, and because *C. sastrei* shows the most extreme caudal morphology, in this section the results are discussed primarily as they relate to the paleobiology of *C. sastrei*. Nonetheless, the functional implications of this study are relevant, to varying degrees, to most known South American abelisaurids

The arguments made by Bonaparte *et al*. 1990 [1] and Mazzetta *et al*. 1998 [11] that *C. sastrei* was a more agile form than other large-bodied theropods is partially supported by this study. The large size of the *M. caudofemoralis* of *C. sastrei* would impart great force to the power strokes of the hind limbs; however, the ridged nature of the caudal series likely reduced turning performance.

The flattened, half-crescent-shaped tips of the caudal ribs of *C. sastrei* overlapped with those directly anterior and posterior in the series, with those of the first caudal vertebra articulating with the ilium [2]. This appears to have resulted in a highly inflexible anterior tail, in terms of both lateral and dorsoventral maneuverability. Recent biomechanical analyses of theropod turning performance have commented on the large rotational inertia that the elongate body-plans of most theropods would impart [24]–[25]. Such studies have likely underestimated the turning abilities of most theropods, because they have assumed that the sum total of a theropod's rotational inertia had to be overcome all at once. Theropods were not laterally stiff, and it is likely that most theropods turned with a more serpentine motion – turning first their heads and necks, then torsos, then hips, and finally, in a sinuous motion, their tails. In the case of *C. sastrei* and the other abelisaurids that shared the interlocking caudal rib morphology, the hips and most of the caudal mass would have been forced to rotate as one unit, and sinuosity would have been minimized. This suggests that *C. sastrei* and it close relatives had a diminished ability to make rapid tight turns, relative to other equivalently sized theropods.

However, the results of the digital muscle reconstruction suggest that what *C. sastrei* lacked in turning ability it may have made up for in overall speed and acceleration. In a sensitivity analysis of bipedal dinosaur running, Bates et *al.* [26] found that locomotive muscle mass and cross-sectional area were the most important factors in estimations of top running speeds. Because the *M. caudofemoralis* was the primary femoral retractor, the large relative mass and corresponding large relative cross-sectional area of the *M. caudofemoralis* of *C. sastrei* would impart exceptional strength to the backwards strokes of the hind limbs. Such a large investment in caudofemoral mass would translate into enhanced locomotive force generation. For an animal as massive as *C. sastrei*, overcoming its own inertia would pose a considerable hindrance to rapid acceleration. The enlarged *M. caudofemoralis* may have provided *C. sastrei* with the raw power necessary for sudden straight-forward sprints and charges.

This investment in locomotive power required a tradeoff in muscle masses. Dorsally tilting the caudal ribs allowed for a larger *M. caudofemoralis,* but, because the neural spines are observably no more elongated than those of most other similarly sized theropods, it also left relatively less space available to be filled by the *M. spinalis* and *M. longissimus*. Both the *M. spinalis* and *M. longissimus* function in mediolateral and dorsoventral tail movement and in maintaining tail stability. While overall tail maneuverability was lost, the interlocking tips of the caudal ribs served to compensate for the diminished epaxial musculature by enhancing tail stability and were perhaps key to allowing the dorsal expansion of the *M. caudofemoralis*. The increased relative stiffness of the anterior portion of the tail likely aided in providing a rigid framework for the large caudofemoral muscles to pull against and likely mitigated energy loss that would have resulted from any lateral or dorsal swing of the tail towards the contracting muscle.

On its own, increased maximum femur retraction force has positive implications for the overall cursorial potential of *C. sastrei*. However, it should be noted that the effect the rigidity of the anterior caudal vertebrae had on locomotive endurance is unclear. On the one hand, in computer simulations of *Allosaurus*, Manning [27] found that a stiff trunk had the potential to store significant elastic energy during dinosaur locomotion. The stiff tail of *C. sastrei* may, therefore, have translated to more spring in its step. On the other hand, undulations of dinosaur tails while walking and running could have facilitated preload stretching of the *M. caudofemoralis*, which also had the potential for great energetic efficiency. The enhanced rigidity in the tail of *C. sastrei* may have limited or altogether prevented anterior tail undulations and the resulting energetic benefits.

### Evolutionary Context

*Carnotaurus sastrei* is currently the youngest known South American abelisaurid and is generally regarded as the most derived in its morphological features [2], [28]. In recent years, a series of older South American abelisaurids have been found and help reveal the rough evolutionary sequence that led to the caudofemoral-dominated tail morphology of *C. sastrei*. *Ekrixinatosaurus novasi* and *Ilokelesia aguadagradensis* (from the lower Cenomanian Candeleros Formation, and the upper Cenomanian Huincul Formation, respectively) are among the oldest known South American members of Abelisauridae [28], [29]. Anterior caudal vertebrae are not known for *I. aguadagradensis*, but in *E. novasi* the caudal ribs have slight dorsal inclinations and expanded tips [28]. The mid-caudal vertebrae of both *E. novasi* and *I. aguadagradensis* have caudal ribs with generally similar morphology. The main difference is that the mid-caudal ribs of *E. novasi* are more posteriorly inclined [28]. In both taxa the mid-caudal ribs extend laterally with slight dorsal inclinations and have strong posterior and anterior projections on the tips, producing a “T-shape” in dorsal view. While these “T-shaped” caudal ribs lack the overlapping and interlocking morphology of *C. sastrei*, the anterior and posterior projections nearly abutted with the next tips in the series and were likely connected by ligaments or other more sturdy tissue. In the caudal vertebrae of *I. aguadagradensis,* the neural spines appear to be reduced in relative dorsoventral height (however, this observation is tenuous, because the incompleteness of the caudal series makes determining the exact position of each vertebra difficult), indicating that the epaxial muscle mass was somewhat reduced, but with no strong evidence of increased relative hypaxial muscle mass.

*Skorpiovenator bustingorryi* [4] is a slightly younger abelisaurid (from the Huincul Formation, Late Cenomanian – Early Turonian). The morphology of the caudal ribs of *S. bustingorryi* closely resembles those of *E. novasi* and *I. aguadagradensis,* but the ribs have notably stronger dorsal inclinations and the anterior projections of the tips of the caudal ribs are more pronounced than the posterior projections [4]. The next youngest South American abelisaurid for which good caudal material is known is *Aucasaurus garridoi* (from the Campanian Rio Colorado Formation) [2]. *A. garridoi* is regarded by many to be the sister taxon to *C. sastrei* [2], [4], [28] (but for an alternative interpretation see Carrano and Sampson [30]). The caudal ribs of *A. garridoi* have a strong dorsal orientation with interlocking tips. *A. garridoi* still has “T-shaped” caudal ribs, but the posterior projections are smaller in relation to the anterior projections.

**Figure 6:**
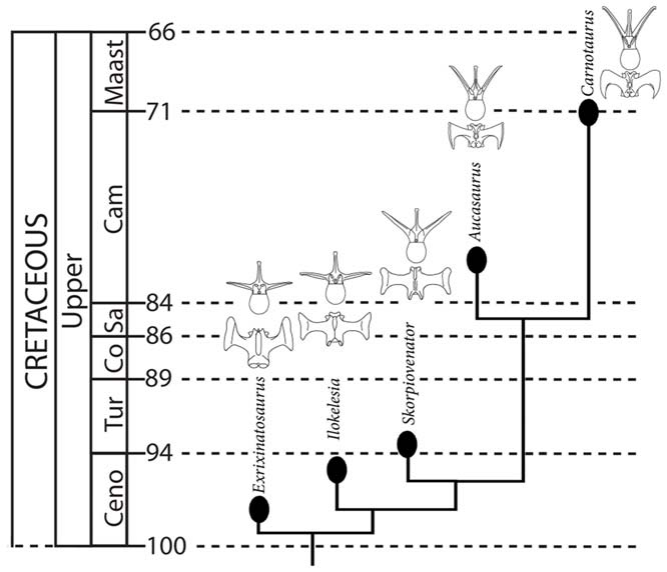
Chronostratigraphy and hypothesized phylogeny of South American Abelisauridae with representative caudal vertebrae for each in anterior and dorsal views. Note: although each taxon is demarked by a separate branching event, given the close geographic and temporal proximities of these taxa, combined with the unlikelihood that multiple other as-yet-unknown large-bodied carnivorous abelisaurids were coexistent, it is probable that some of these taxa have a direct anagenetic relationship with others.

The phylogeny of the Abelisauridae has been the subject of much analysis, debate, and uncertainty. Based on the caudal morphology and the chronology of taxa, the overall evolutionary sequence of South American abelisaurids seems to have been: 1) slight dorsal inclining of the caudal ribs and the development of anterior and posterior projections on the tips of the caudal ribs, which increased rigidity in the caudal series and diminished the need and functional value of the caudal epaxial musculature (seen in *E. novas* and *I. aguadagradensis*); 2) the gradual increase in the dorsal inclination of the caudal ribs (*S. bustingorryi)* and corresponding dorsal expansion and increase in total mass of the *M. caudofemoralis*; 3) a further increase in rigidity accomplished through true interlocking caudal ribs (*A. garridoi*) and continued caudofemoral dorsal expansion; and, 4) maximized rigidity through crescent-shaped, tightly interlocking rib morphology (*C. sastrei*). The evolutionary sequence we suggest is summarized in Figure 6.

This phylogeny should not be misinterpreted as a well substantiated cladistic conclusion. It is instead a tentative hypothesis derived solely from two lines of evidence (caudal morphology and chronological sequence). It is offered here with the hope that it will be validated or invalidated by future studies, and with the encouragement that subsequent cladistic analyses of Abelisauridae (which have previously been heavily reliant on cranial characters) take into more thorough consideration the morphology of the caudal vertebral series.

Regardless of the true phylogeny, increased *M. caudofemoralis* mass and caudal rigidity appear to be characteristic of later South American abelisaurids. This result, and its inferred relation to relative cursoriality, is consistent with previous observations on the limb proportions of South American abelisaurids [13], which reported longer and more gracile limbs in *Carnotaurus*, *Aucasaurus*, and *Skorpiovenator* than in earlier genera. These results also show a strong contrast between the late abelisaurids of South America and those known from the rest of Gondwana – which have primitive caudal morphology and short, stocky hind-limb proportions [12], [14], [30]. In particular, these results conflict with previous conclusions that the Malagasy abelisaur *Majungasaurus crenatissimus* and *C. sastrei* were more closely related to each other than ether were to any of the other South American taxa [31]–[33].

### Conclusions

**Figure 7:**
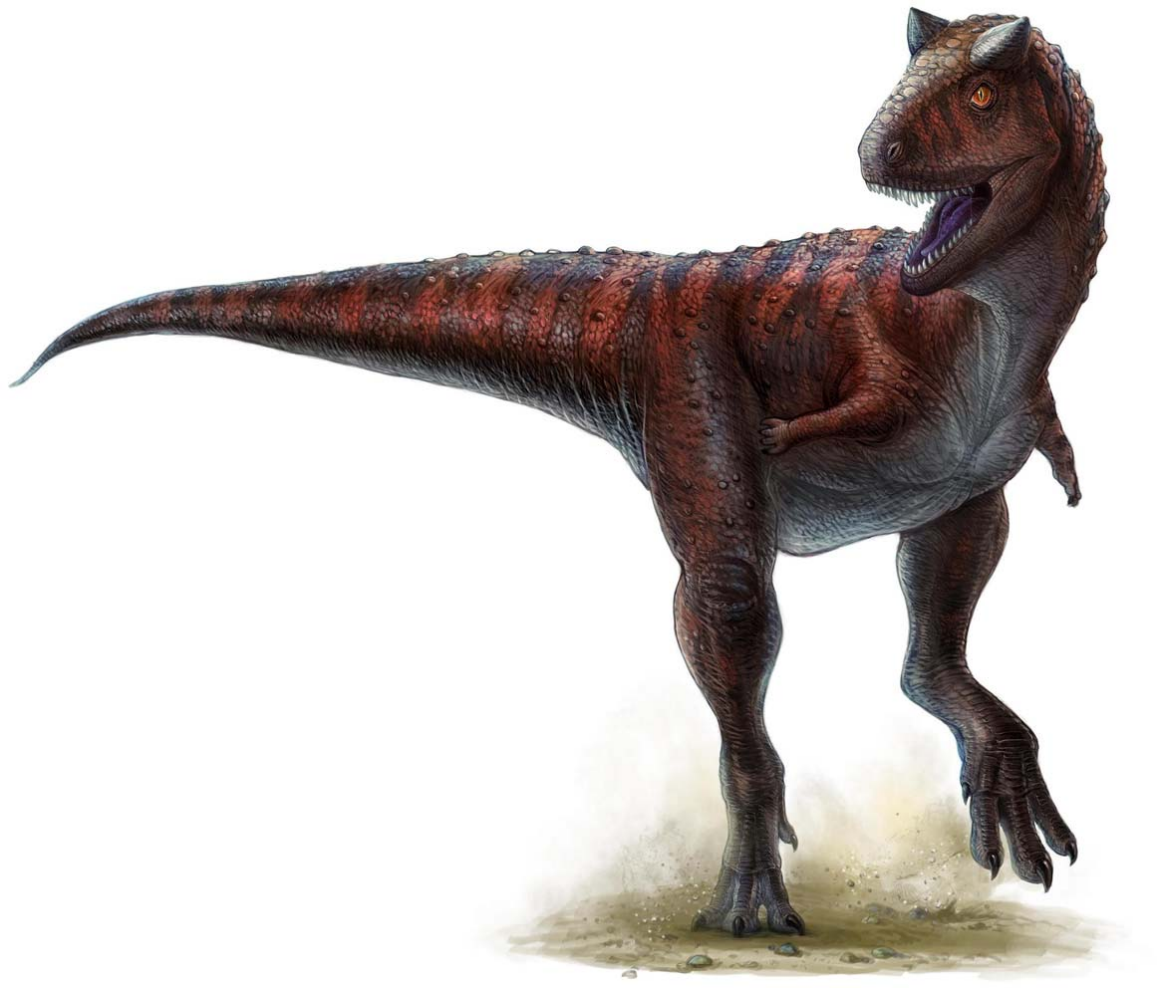
Life restoration of a sprinting *Carnotaurus sastrei*, by Lida Xing and Yi Liu. Illustration shows laterally expansive and appropriately large caudal musculature.

In his examination of the *M. caudofemoralis* of theropod dinosaurs, Gatesy [17] posited a general trend of relative caudofemoral muscle size reduction throughout the whole of theropod evolution. As shown by Gatesy [17], a strong trend toward reduced caudofemoral mass can be seen in the lineage leading to modern birds. However, the unique caudal vertebrae morphology of *Carnotaurus sastrei* and its close relatives offers a dramatic counterexample. The development of interconnecting caudal ribs, each with a strong dorsal inclination, enabled an exceptionally large *M. caudofemoralis*. This would have made *Carnotaurus sastrei* a powerful sprinter – perhaps among the fastest of the large bodied theropods (see Fig. 7). Consideration of these morphological differences in a stratigraphic context indicates a pattern of increased caudofemoral mass and cursorial potential throughout the evolutionary history of the Abelisauridae of South America. During at least the early portion of this history, abelisaurids coexisted with another clade of predatory dinosaurs: carcharodontosaurids. The carcharodontosaurids of South America (including *Giganotosaurus carolinii*, *Mapusaurus roseae*, and *Tyrannotitan chubutensis*) were among the largest of all theropods, and obtained body sizes much greater than that of any known abelisaurid [13]. It has been argued that the extreme size of these carcharodontosaurids allowed them to hunt the even larger South American titanosaur sauropods [34]. The cursorial tail morphology of South American abelisaurids may have arisen to help in avoiding potential carcharodontosaurid predators and/or supported niche partitioning by allowing abelisaurids to specialize in the pursuit and capture of smaller prey, such as ornithopods.

## Methods

Digital skeletal and muscle models of *Carnotaurus sastrei* were created following procedures shown to be accurate for modern taxa [22]. All models were created using the digital modeling program Rhinoceros® [35].

### Skeleton

MACN-CH 894 includes only the first six caudal vertebrae and an isolated fragment interpreted by Bonaparte *et al.* [1] as possibly belonging to caudal vertebra 12. In the digital model, the first six caudal vertebrae were sculpted based on measurements made on LACM 127704 (a cast of MACN-CH 894). To ensure accuracy, measurements of LACM 127704 were compared to those published in Table 2 of Bonaparte *et al*. 1990 [1], and were found to be reliable (see Table S1).

The remaining vertebrae had to be digitally sculpted based on published measurements and illustrations of other abelisaurid material and scaled to fit. Caudal vertebrae 7–13 were modeled based on MCF-PVPH-236, the holotype of *Aucasaurus garridoi* [2]. The anterior caudal vertebrae of *A. garridoi* show a morphology similar in most respects to that of *Carnotaurus* (although the caudal ribs of *Aucasaurus* are not as dorsally inclined and lack the distinctive crescent shape), and the two theropods are considered by most authors to be sister taxa [2], [4], [28]. Thus, the posterior portion of the created “*Carnotaurus*” model may be more representative of a generalized carnotaurine, but the model does include all the known advanced caudal morphology of *C. sastrei*, with the region modeled after *A. garridoi* modified to fit the proportions of the anterior sequence and scaled to conform to the large body size of *C. sastrei*.

Deducing the shape of the more posterior vertebrae requires greater speculation. Fortunately, beyond caudal vertebra 13, the vertebrae and associated muscles are so diminished in size that reasonable variation in their shape and total number can only have minimal effects on the calculations of muscle mass (see Fig. S1, S2 and Table S2). Caudal vertebrae 14–25 were based on those of *Majungasaurus crenatissimus* (FMNH PR 2100) – the only abelisaurid for which a reasonably good series of posterior caudals has been described. Based on the trend of vertebral size reduction observed in the more anterior vertebrae, the series is estimated to have ended at caudal vertebra 42, and the remaining 17 vertebrae were based on those of *Ceratosaurus nasicornis* (USNM 4735). It is assumed that in the more posterior vertebrae the caudal ribs successively assumed a more typical, horizontal orientation. This assumption seems reasonable, given that in other theropods with slightly dorsally oriented caudal ribs on the anterior caudal vertebrae (such as *Allosaurus fragilis* and *Ceratosaurus nasicornis*), the caudal ribs of the posterior caudals lose all dorsal inclination.

In the description of the type specimen, the chevrons of *C. sastrei* were reconstructed with strong posterior angulations [1]. The chevrons of most non-avian theropods show some degree of posterior orientation, but none are as extreme as those depicted for *C. sastrei* (Fig. 38 of Bonaparte *et al*., 1990 [1]). The articulated caudal series preserved for *Aucasaurus garrido* shows chevrons with posterior angulations relative to the axis of the caudal vertebrae, but the chevrons are still less posteriorly inclined than in the original depiction of *C. sastrei*. In the digital model, the anterior chevrons have been inclined to angles consistent with those seen in *A. garrido*. This more conservative chevron orientation is also consistent with the angles of the chevron indentations preserved with the skin impressions of MACN-CH 894. A possible explanation for the discrepancy between the chevron angulations proposed here and those proposed by Bonaparte *et al*. [1] is that the single well-preserved chevron of MACN-CH 894, which was tentatively identified by Bonaparte *et al*. 1990 [1] as chevron number four, was in fact chevron number one or two. The first two chevrons of crocodiles and many modern reptiles are more strongly inclined posteriorly than the other chevrons in the series, and this is also the case in numerous theropod genera [36], including *A. garrido* (see Fig. 2 in Coria *et al*., 2002 [2]).

**Figure 8:**
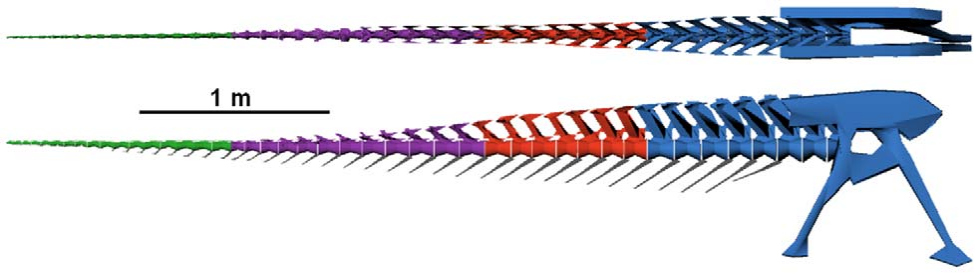
Digital model of the tail of *Carnotaurus sastrei*. Blue vertebrae modeled after *Carnotaurus sastrei* (MACN-CH 894), red vertebrae modeled after *Aucasaurus garridoi* (MCF-PVPH-236), purple vertebrae modeled after *Majungasaurus crenatissimus* (FMNH PR 2100), and green vertebrae modeled after *Ceratosaurus nasicornis* (USNM 4735).

The digital reconstruction assumes that the total number of caudal vertebrae in the tail of *C. sastrei* was 42, that the posterior caudal ribs became gradually less dorsally inclined and terminated at caudal vertebrae 26, and that the orientations of the chevrons were similar to those seen in *A. garrido* (see Fig. 8).

### Epaxial Musculature

Following the terminology scheme established in Persons and Currie 2011 [22], the epaxial tail musculature is divided into two major muscle sets: the dorsal *M. spinalis* and the ventral *M. longissimus*. Throughout the caudal series, the *M. spinalis* inserts onto the tips and lateral surfaces of the neural spines. Anteriorly, the *M. longissimus* inserts onto the lateral surfaces of the neural arches and the dorsal surfaces of the caudal ribs. More posteriorly, after the termination of the caudal ribs, the *M. longissimus* only inserts onto the lateral surfaces of the neural arches (see Persons and Currie [22], for a complete review of theropod caudal muscle insertions). The septum that divides the *M. spinalis* from the *M. longissimus* leaves no clear insertion scar, but is reconstructed based on the morphology observed in dissections of modern sauropsids.

### Hypaxial Musculature

The hypaxial tail muscles consist of two large muscle sets: the *M. ilio-ischiocaudalis* and the *M. caudofemoralis* [6]. The *M. ilio-ischiocaudalis* is composed of multiple myomere series and can be subdivided into the *M. iliocaudalis*, which originates from the ilium, and the *M. ischiocaudalis*, which originates from the ischium. The *M. ilio-ischiocaudalis* extends posteriorly to the tip of the tail. The *M. caudofemoralis* is composed of long uninterrupted muscle fibers and can be subdivided into the *m. caudofemoralis brevis*, which fills the brevis fossa, and the *m. caudofemoralis longus*, which tapers posteriorly. Both the *m. caudofemoralis brevis* and the *m. caudofemoralis longus* insert onto the fourth trochanter of the femur, and together serve as the primary limb retractor [17]–[18].

Gatesy 1990 [17] argued that the posterior tip of the *M. caudofemoralis* was correlated with the termination of the caudal ribs. Persons and Currie 2011 [22] identified a scar on the broad haemal spines of some well-preserved theropod specimens as the insertion of the septum that separated the *M. ilio-ischiocaudalis* from the *M. caudofemoralis*. This scar could, therefore, be used to identify the posterior tip of the *M. caudofemoralis*. Well preserved haemal spines from the region of the tail where the posterior terminus of the *M. caudofemoralis* would be expected have yet to be described for *C. sastrei* or any of its close relatives. In *C. sastrei*, the position of the posterior tip must be inferred, based on the point of caudal rib termination, which must in turn be inferred from the general trend in caudal rib reduction seen in the anterior vertebrae and from the caudal rib termination point of the distantly related *Majungasaurus crenatissimus*.

Using comparisons with modern reptiles, Wilhite [37] and Persons and Currie [22] argued that anterior to its posterior termination, the *M. caudofemoralis* of dinosaurs inserted across the full lateral surfaces of the centra and chevrons. Contrary to numerous depictions (e.g. [15], [20]), the *M. caudofemoralis* did not insert onto the ventral surfaces of the caudal ribs (which are strictly insertions of the *M. ilio-ischiocaudalis*).

**Figure 9:**
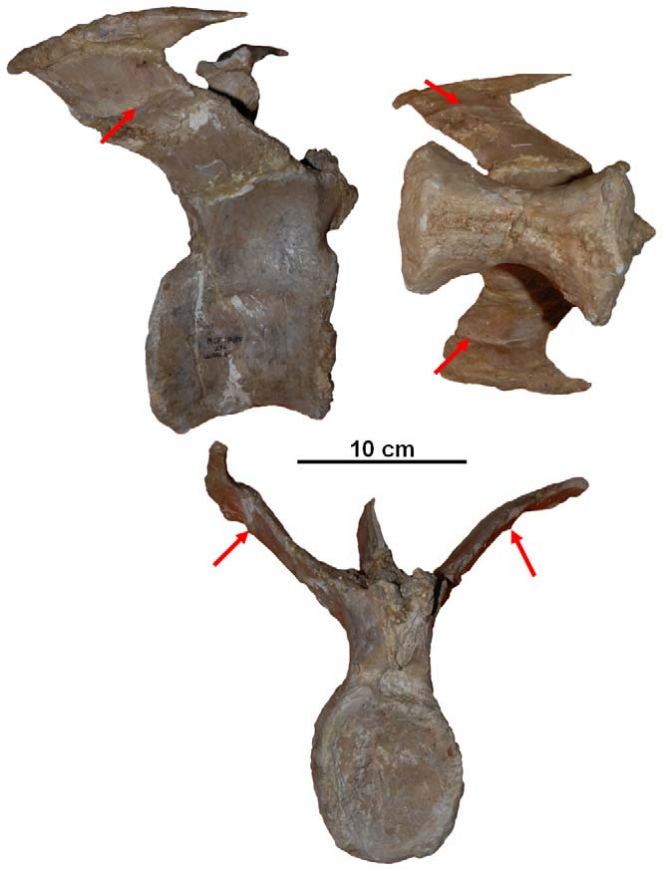
Caudal vertebra 4 of *Aucasaurus* (MCF-PVPH-236) in (A) lateral, (B) dorsal, and (C) anterior view. Arrows indicate the *M. ilio-ischiocaudalis*/*M. caudofemoralis* septum scars.

**Figure 10:**
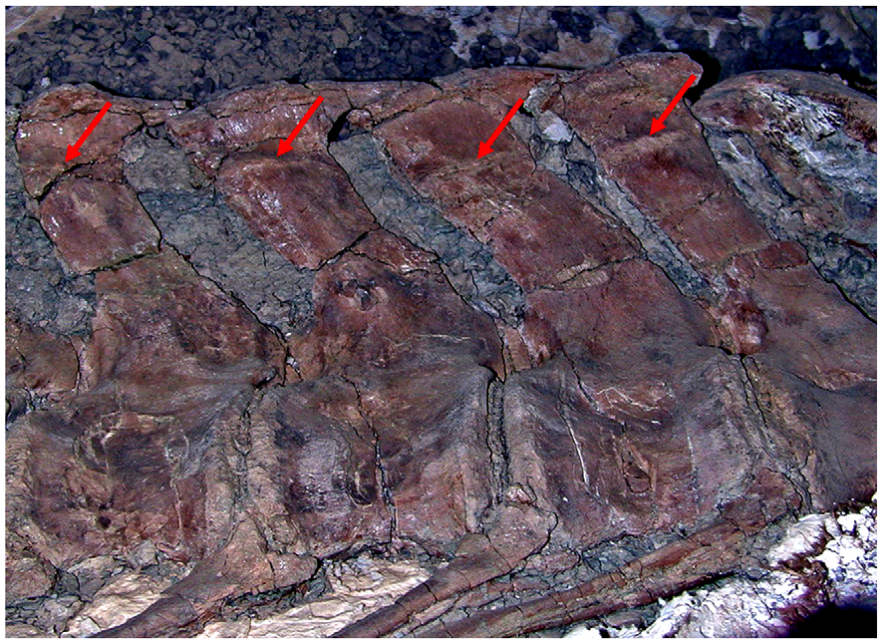
Caudal vertebra 1–4 of *Aucasaurus garridoi* (MCF-PVPH-236) in lateral view. Arrows indicate the sequence of *M. ilio-ischiocaudalis*/*M. caudofemoralis* septum scars.

However, Persons and Currie [22] suggested that the nearly vertical caudal ribs of some advanced abelisaurids were possible exceptions. The well preserved anterior caudal series of *Aucasaurus garrido* offers strong evidence that this was indeed the case, and that the caudal ribs of advanced South American abelisaurids were insertion surfaces for both the *M. ilio-ischiocaudalis* and the *M. caudofemoralis*. The ventral surface of each caudal rib of *A. garrido* shows a narrow anteriorposteriorly directed scar (Fig. 9,10) that strongly resembles the haemal spine scar interpreted in Persons and Currie [22] as the insertion of a septum in other theropods.

**Figure 11:**
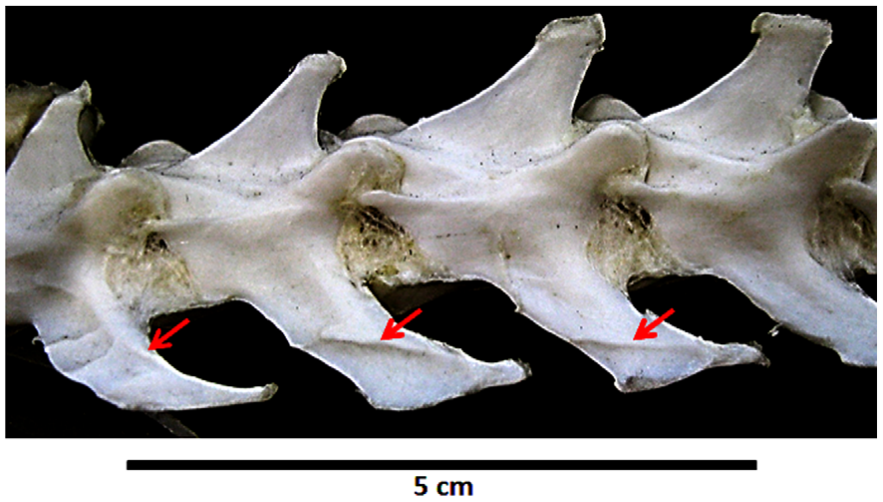
Example of muscle septa scars. Arrows point to scars on the anterior most lumbar vertebra of *Felis catus* that demark the boundary between two epaxial muscle insertions (right lateral view).

Carrano et al. [12] interpreted the caudal rib scars of *A. garrido* as sutures between fused caudal ribs and caudal transverse processes. This interpretation is here disfavored, because the scars do not form continues rings around the caudal ribs, but are instead pronounced only on the ventral surfaces. The scars are also morphologically dissimilar to typical sutures, being substantially distended from the surrounding bone surface and tapered to form central keels. However, the scars are morphologically similar to vertebral septum insertion scars observable in modern animals, such as on the dorsal surfaces of lumbar transverse-processes, which commonly demark boundaries between epaxial muscle in mammals (Fig 11) (pers. obs.).

These caudal rib scars are here interpreted as marking the dorsal insertion of the *M. ilio-ischiocaudalis*/*M. caudofemoralis* septum. Using these scars as a guide, it is possible to reconstruct how far dorsally the *M. caudofemoralis* extended across the caudal ribs of *A. garrido*, and, by analogy, approximately how far the *M. caudofemoralis* extended across the caudal ribs of *C. sastrei* (Fig. 5).

### Musculature Modeling

Following methods similar to those used by Arbour [20], Allen *et al.* [21], and Mallison [23], two muscle reconstructions were created. One is a conservative reconstruction that is comparable with those created in Persons and Currie [22]. The other is a robust reconstruction.

In the conservative model, the whole of the epaxial musculature was reconstructed by extending an arc in dorsoventral cross-section from the tips of the neural spines to the tips of the caudal ribs; anterior to its tapering, the *M. caudofemoralis* was reconstructed by extending an arc in dorsoventral cross-section from its attachment site on the ventrolateral surface of the caudal ribs to the ventral tip of the chevrons. The *M. ilio-ischiocaudalis* was reconstructed anterior to the tapering point of the *M. caudofemoralis*, by extending an arc in dorsoventral cross-section from the lateral tips of the caudal ribs to below the ventral tips of the chevrons, and, posterior to the tapering point of the *M. caudofemoralis* by extending an arc in dorsoventral cross-section from the ventral boundaries of the neural arches to the ventral tips of the chevrons. Anteriorly, the arc of the *M. ilio-ischiocaudalis* maintained a consistent thickness equal to the distance between the reconstruction of the *M. caudofemoralis* and the ventrolateral edge of the caudal ribs. Note that this method of conservative reconstruction is not synonymous with the “traditional elliptical” reconstruction method described in Allen *et al.* [21], and in the conservative model the dimensional extents of the caudal musculature greatly exceeds that of the caudal osteology.

In the robust model, the epaxial musculature arc was assumed to extend beyond the neural spines and caudal ribs by 25% and 75%, respectively. The *M. caudofemoralis* was reconstructed with a laterally oblong shape; and the *M. ilio-ischiocaudalis* was thickened such that the lateral extreme of its arch extended beyond that of the anterior caudal ribs by 400%. Like the conservative model, the robust model assumes that no large fat deposits were present in the tail, although in modern sauropsids the tail is a common site of fat storage. In modern crocodilians, a thick layer of fat is often deposited between the *M. caudofemoralis* and the *M. ilio-ischiocaudalis* [38]. The proportions used in the robust model conform to those observed in the girthy anterior-most caudal regions of modern reptiles [21], [23], [38], with fat deposits removed.

The portion of the *M. caudofemoralis* reconstructed in both the conservative and the robust model corresponds to the *m. caudofemoralis longus*. The *m. caudofemoralis brevis* was not modeled. Instead, the mass of the *m. caudofemoralis brevis* was estimated by measuring the volume of the brevis fossa. Because the *m. caudofemoralis brevis* is completely capped by the brevis fossa, the size of the *m. caudofemoralis brevis* is far less speculative, and its contribution to the final mass estimations was not varied between the robust and conservative results.

## Supporting Information

Figure S1 **Long tail model of** ***Carnotaurus sastrei*** **(MACN-CH 894) reconstructed to test for muscle mass variation resulting from uncertain posterior tail form.** Reconstruction assumes five additional posterior vertebrae and posterior chevrons and caudal ribs that decrease in size more gradually. Muscle reconstruction follows the conservative method. (A) Digital reconstruction of the caudal and pelvic skeleton with *M. caudofemoralis longus* (red). (B) Complete digital reconstruction, with epaxial musculature (orange) and *M. ilio-ischiocaudalis* (pink) added.
(TIF)

Figure S2 **Short tail model of** ***Carnotaurus sastrei*** **(MACN-CH 894) reconstructed to test for muscle mass variation resulting from uncertain posterior tail form.** Reconstruction assumes five fewer posterior vertebrae and posterior chevrons and caudal ribs that decrease in size more rapidly. Muscle reconstruction follows the conservative method. (A) Digital reconstruction of the caudal and pelvic skeleton with *M. caudofemoralis longus* (red). (B) Complete digital reconstruction, with epaxial musculature (orange) and *M. ilio-ischiocaudalis* (pink) added.
(TIF)

Table S1 **Measurements of LACM 127704 (a cast of Carnotaurus sastrei MACN-CH 894).** Direct measurements of MACN-CH 894 from Bonaparte et al. [1] included for comparison. All measurements given in millimeters.
(XLSX)

Table S2 **Comparison of mass estimations from the test of muscle mass variation resulting from uncertain posterior tail form.** Results indicate total tail muscle mass is potentially affected by less than 7%.
(XLSX)
