# Supplementary figures and images for: Dinosaur Speed Demon: The Caudal Musculature of Carnotaurus sastrei and Implications for the Evolution of South American Abelisaurids
Source: PLoS One. 2011 Oct 17;6(10):e25763. doi: 10.1371/journal.pone.0025763 (PMC3197156; doi:10.1371/journal.pone.0025763)

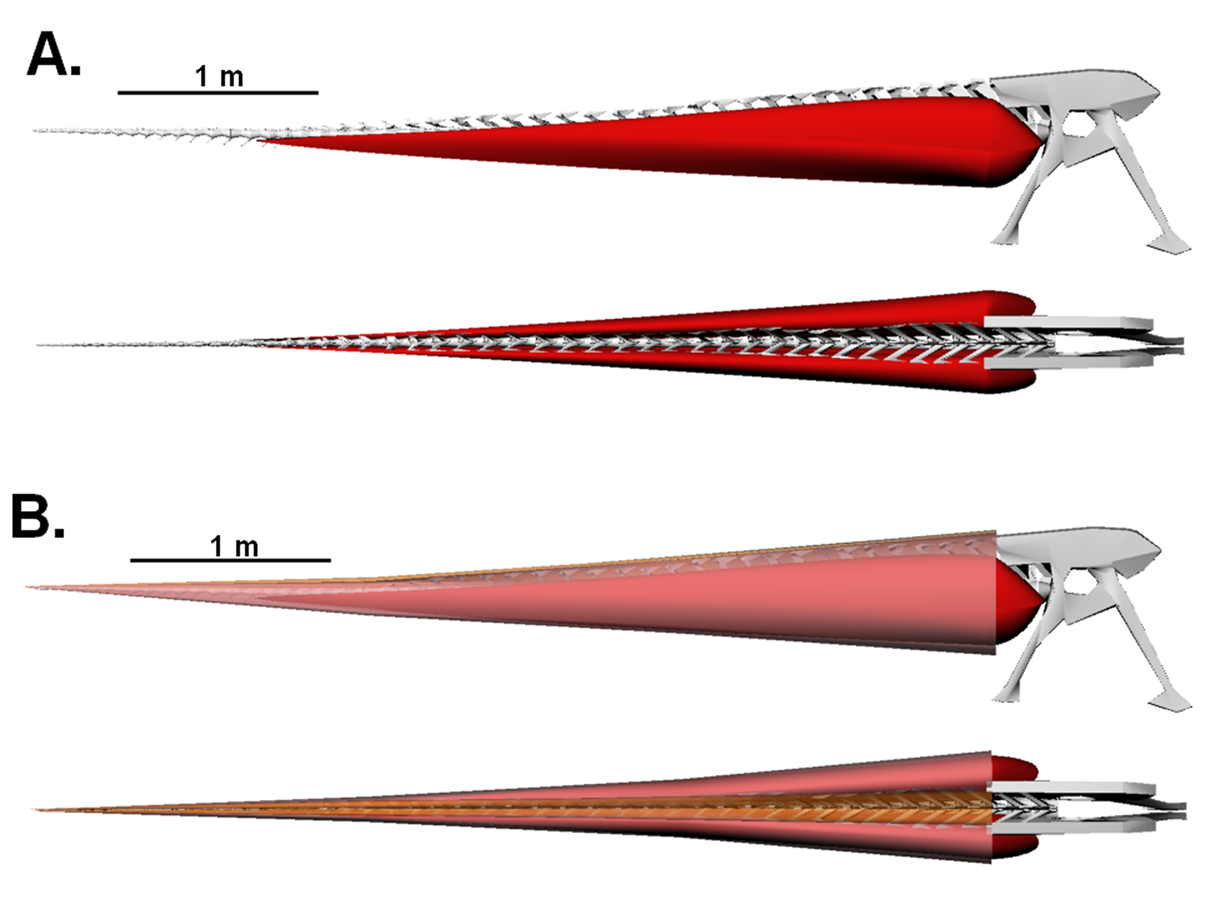

Supplement: Figure S1 — Long tail model of Carnotaurus sastrei (MACN-CH 894) reconstructed to test for muscle mass variation resulting from uncertain posterior tail form. Reconstruction assumes five additional posterior vertebrae and posterior chevrons and caudal ribs that decrease in size more gradually. Muscle reconstruction follows the conservative method. (A) Digital reconstruction of the caudal and pelvic skeleton with M. caudofemoralis longus (red). (B) Complete digital reconstruction, with epaxial musculature (orange) and M. ilio-ischiocaudalis (pink) added. (TIF) [file pone.0025763.s001.tif]

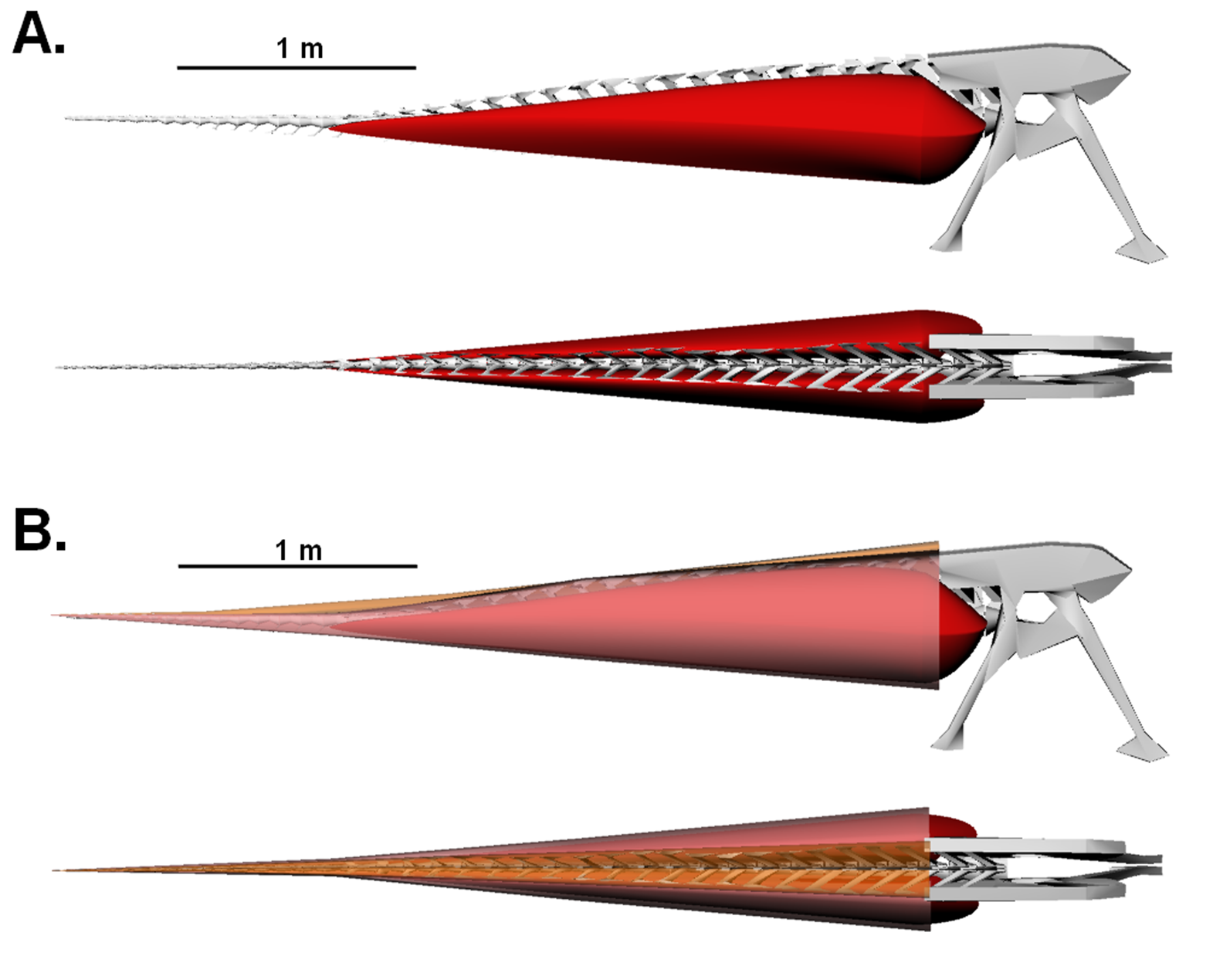

Supplement: Figure S2 — Short tail model of Carnotaurus sastrei (MACN-CH 894) reconstructed to test for muscle mass variation resulting from uncertain posterior tail form. Reconstruction assumes five fewer posterior vertebrae and posterior chevrons and caudal ribs that decrease in size more rapidly. Muscle reconstruction follows the conservative method. (A) Digital reconstruction of the caudal and pelvic skeleton with M. caudofemoralis longus (red). (B) Complete digital reconstruction, with epaxial musculature (orange) and M. ilio-ischiocaudalis (pink) added. (TIF) [file pone.0025763.s002.tif]
